# P07 Beyond *Mycobacterium ulcerans*: secondary bacteria contribute to antimicrobial resistance in Buruli ulcer treatment

**DOI:** 10.1093/jacamr/dlag102.013

**Published:** 2026-06-26

**Authors:** Yaa Yeboaa Asare, Jennifer Morgan, Sandra Benin, Susana Debrah, Mabel Sarpong Duah, Charles Quaye, Lydia Mosi

**Affiliations:** West African Centre for Cell Biology of Infectious Pathogens (WACCBIP), University of Ghana, Accra, Ghana; West African Centre for Cell Biology of Infectious Pathogens (WACCBIP), University of Ghana, Accra, Ghana; Mississippi State University, Starkville, MS, USA; West African Centre for Cell Biology of Infectious Pathogens (WACCBIP), University of Ghana, Accra, Ghana; School of Public Health, University of Ghana, Accra, Ghana; Noguchi Memorial Institute for Medical Research, University of Ghana, Accra, Ghana; West African Centre for Cell Biology of Infectious Pathogens (WACCBIP), University of Ghana, Accra, Ghana

## Abstract

**Background and objectives:**

Buruli ulcer (BU) is a skin necrotizing disease caused by *Mycobacterium ulcerans*. It is characterized by painless lesions and treatment is by antimycobacterial agents. Previously, it was believed that mycolactone, the main virulence factor of the bacteria was highly bactericidal rendering other bacteria unable to survive in the lesions. However, some recent studies have proven this to be false. The presence of secondary bacteria in these lesions may lead to pain, delay in wound healing and antimicrobial resistance-related treatment inefficacy. Culture-dependent techniques have been relied on to depict the microbial diversity of Buruli ulcer lesions. However, this is inadequate as unculturable bacteria are unaccounted for and slow-growing culturable bacteria are outcompeted. Some studies have addressed this by using culture independent tools such as targeted 16S rRNA amplification and sequencing. However, the small sample sizes and inadequate controls require additional work which this study achieves by using metagenomic sequencing on a larger sample size and controls from BU-endemic communities in Ghana. In addition, not many studies have investigated into detail the role these secondary bacteria play on antimicrobial resistance to the treatment regimen for BU, which study also overcomes.

**Methods:**

Prospective swab samples were collected from BU-like lesions in two endemic areas in Ghana. Real time polymerase chain reaction (qPCR) targeting the IS2404 gene was performed for BU confirmation. By employing Metagenomic sequencing, the microbiome profile of the lesions was determined and associations. Simultaneously, using culturing and 16S rRNA conventional PCR, secondary bacteria were isolated and characterized from the samples. After which the antimicrobial profiles of the isolates were investigated by testing against selected antibiotics that are the conventional treatment drugs for BU and others that are not.

**Results:**

Out of the 67 samples collected from 63 participants, 11 tested positive for BU which were considered test samples and the remaining 56 considered as controls. Up to 44 bacterial genera were identified from sequencing data as compared to the 13 obtained from culturing. Bacterial diversity within these individual samples was relatively low, likely because of highly selective conditions like mycolactone toxin. In, addition, the microbial population in the lesions also showed that the samples are very similar in composition (species present) when subjected to a pair-wise comparison. *Staphylococcus aureus* was the most dominant isolate among the 61 isolates obtained. Upon subjecting them to the commonly prescribed BU treatment antibiotics; rifampicin, clarithromycin, Kanamycin, and Streptomycin, almost all the isolates were resistant barring a little over 10 % which were susceptible to Rifampicin and <5 % which showed intermediate susceptibility to clarithromycin. It was observed that there was considerable resistance to the other antibiotics tested; penicillin/streptomycin and ciprofloxacin.

**Conclusions:**

This study has shown that there is varying bacterial diversity in BU lesions and normal skin commensals such as *S. aureus* can transition into opportunistic bacteria thereby causing pain and recurring secondary bacterial infections that contribute to antimicrobial resistance and consequently delay in wound healing. This knowledge is pivotal as it contributes to steps to review the current BU treatment regimens.

